# Unleashing NK cells for cancer immunotherapy in lung cancer: biologic challenges and clinical advances

**DOI:** 10.1186/s13046-025-03503-7

**Published:** 2025-08-23

**Authors:** Quinlan McLaughlin, Dorothy K. Sojka, Kathleen Kennedy, Sytse J. Piersma, Nan Sethakorn

**Affiliations:** 1 Stritch School of Medicine, Loyola University Chicago, Maywood, IL USA; 2 Department of Microbiology and Immunology, Loyola University Chicago, Maywood, IL USA; 3 Loyola University Chicago Cardinal Bernardin Cancer Center, Maywood, IL USA; 4 Department of Medicine, Division of Rheumatology, Washington University St. Louis School of Medicine, St. Louis, MO USA; 5 Washington University St. Louis Siteman Cancer Center, St. Louis, MO USA

**Keywords:** Natural killer cells, Non-small cell lung cancer, Immunotherapy

## Abstract

Natural killer (NK) cells are a crucial part of the innate immune system and serve as an important effector for killing tumor cells through direct cytolytic activity or immunomodulatory signaling to T cells and antigen presenting cells. NK cells are correlated with increased tumor control and better overall patient survival across various types of cancers including non-small cell lung cancer (NSCLC). Despite their promising potential for anti-tumor killing, NK cell function is often diminished within the tumor microenvironment. There are many factors that lead to decreased tumor-infiltrating NK cell killing, including immunoinhibitory factors from tumor cells and resident tissues, acquired immune tolerance, NK cell exhaustion, and the hypoxic state of the tumor microenvironment. Unleashing NK cell activity therefore has high potential to create a new class of immunotherapy that could combat both primary and acquired resistance to current checkpoint inhibitors. In this review we discuss mechanistic details of NK cell tumor killing, NK cell immunosuppression, and gaps in knowledge regarding highly complex microenvironment-specific effects on NK cell function. We also discuss the promise and limitations of emerging NK-cell based therapeutic strategies.

## Background

Lung cancer remains the leading cause of cancer related deaths, and despite the transformative effect of immunotherapy for non-small cell lung cancer (NSCLC), most patients develop acquired resistance to these treatments [1]. Immune checkpoint blockade that activates CD8 + T cells is the standard of care for patients with metastatic non-small cell lung cancer (NSCLC) lacking actionable genomic alterations (such as EGFR and ALK alterations) [1, 2]. Long-term survival in patients with metastatic NSCLC approximates 30% in subsets of patients responsive to anti PD1/PD-L1 inhibitors, however most patients do not derive long term benefit [1]. Multiple mechanisms underlie this phenomenon, and promising approaches include activating additional immune cell subsets such as Natural killer (NK) cells. NK cells are part of the innate immune system and exert crucial functions in anti-tumor immunity through both direct tumor cell lysis and amplification of immunomodulatory signaling to T cells and antigen presenting cells. Therapies based on augmentation of NK cell functions are a new frontier in immunotherapy [3]. In this review, we consolidate studies on NK cell biology, NK cell (dys)function in anti-cancer immunity, opportunities for therapeutic targeting, and the current state of clinical investigation into NK cell-based therapies specifically relevant to lung cancer.

### NK cell biology

Natural killer (NK) cells are part of the innate immune system responsible for surveillance and clearance of non-self cells. NK cell cytotoxic activity is tightly regulated by a balance of activating and inhibitory receptors, in order to limit uncontrolled NK cell killing. Expression of activating receptor ligands are frequently induced by cell stressors, such as malignant transformation and virally-infected cells. A comprehensive summary of NK cell inhibitory and activating ligands was summarized by Vivier, et al. [4].

Classically, NK cells have been studied in the human peripheral blood where they have been phenotypically characterized as CD3-CD56dimCD16 + cells. To date, two functionally and phenotypically distinct subsets of NK cells are classified by the differential expression of CD56 and CD16 on the cell surface; CD56dimCD16 + and CD56brightCD16dim/- [5, 6]. The CD56dimCD16 + are highly cytotoxic and comprise 90–95% of the NK cells found in peripheral blood, lymph nodes, spleen [5]. High expression of CD16, the low-affinity Fc receptor, allows for antibody-dependent cellular cytotoxicity (ADCC), whereas low CD16 expression abolishes ADCC [6]. CD56dimCD16 + cells express inhibitory killer cell immunoglobulin-like receptors (KIR), activating receptors including NKG2D, DNAM1, and the natural cytotoxicity receptors NKp44, NKp30 and NKp46 [4]. Cytolytic NK cells can induce target cell death through directed release of lytic granules or by inducing death receptor mediated apoptosis via the expression of Fas ligand or TNF-related apoptosis inducing ligand (TRAIL). NK cell recognition of a target cell activates the polarized exocytosis of the lytic granules, specialized organelles containing pore-forming molecules perforin and serine protease granzymes, that aid target cell death. While granule mediated cytotoxicity is fast, death receptor mediated apoptosis requires more time [7]. Rebuffet, et al., utilized a combination of single cell RNAseq and cellular indexing of transcriptomes and epitopes by sequencing (CITE-seq) to add a third group of peripheral blood NK cells, which encompasses adaptive NKG2C + NK cells [8]. This population shows high expression of NKG2C and intermediate expression of cytotoxic markers, and was also identified in multiple tumor tissues including lung, colon cancer, head and neck squamous cell carcinoma, nasopharyngeal carcinoma, and prostate adenocarcinoma [8]. This population can be expanded in response to acute cytomegalovirus infection [9]. However, their role in anti-tumor immunity remains largely unexplored.

CD56bright CD16dim/- NK cells are abundant cytokine producers, less cytotoxic, and are predominant in secondary lymphoid tissues. This is likely because this subset expresses high levels of CCR7 and L-selectin, both required for cellular trafficking [6, 10]. Tissue NK cells express cell surface receptors (such as CD49a, CD69, and CD103) unique to each tissue, and exert different functions specific to their microenvironments and distinct from peripheral NK cells [5, 11]. Tissue-resident NK cells are weakly cytotoxic, but they produce several immunoregulatory cytokines, such as interferon-γ (IFN-γ), tumor necrosis factor-α (TNF- α), granulocyte–macrophage colony-stimulating factor (G-MCSF) [5]. In NSCLC, activation of tissue-resident NK cells were reported to have an exhausted phenotype (CD69 + CXCR6+), highlighting their noncytotoxic function resulting in poor anti-tumor immunity [12].

The development of NK cells primarily occurs in the bone marrow but further differentiates in tissues, and NK cell function is associated with tissue distribution and localization [5, 6]. Besides established cytolytic or tissue-resident NK cells, evidence suggests other subtypes of NK cells may exist, each defined by different surface molecules and functions [13, 14]. For example, a subset of HLA-DR + NK cells have been described to have weak antigen-presenting ability in vitro [15]. Similar functional cells have been observed in mice, and HLA-DR + NK cells were also identified in NSCLC patient tumors [15]. The “helper” NK (NKh) cell differentiation pathway is a subset of CD56 + NK cells that could be induced to express CD83+, CCR7+, and CD25+, common surface markers on mature dendritic cells (DCs) [16]. IL-2 activated NK cells promote the differentiation of mature type-1 polarized DCs (DC1s), these cells display high anti-melanoma activity as they are capable of inducing Th1 and CTL responses in T-cells [17]. This effect is contact dependent, but the secretion of IFN-γ and TNF-α from NK cells contributes to DC maturation as well [18]. A regulatory function of NK cells has been described in mouse models with relevance in controlling autoimmunity, and suggested the concept of regulatory NK cells (NKregs, akin to immunosuppressive Tregs) [6, 19, 20]. For example, DC-activated NK cells exhibited the ability to kill immature DCs in vitro, and postulated to serve as a negative feedback loop [21]. In NSCLC, NK cells isolated from a syngeneic Lewis lung carcinoma (LLC) model secreted CCL22 that recruited Tregs into the tumor [22]. Picard, et al., demonstrated a subtype of CD56dim/CD16- NK cells circulating in blood from patients with NSCLC that produced regulatory cytokines [23]. While there are many studies describing these subtypes, several of them were performed in murine models and thus additional investigation of patient tumors will be necessary to determine clinical relevance. Furthermore, it is unknown whether these cells represent unique NK subsets with distinct in vivo functions, or whether inducible expression of markers indicate transient differentiation states of tissue-resident NK cells. Taken together, NK cell function is context dependent and consideration should be given to NK cell tissue localization and subset heterogeneity in the analysis of NSCLC tumors.

As aforementioned, NK cells recognize target cells through activation and inhibitory receptors. NK cell activating receptors include NKG2D, whose ligands (NKG2D-L) can frequently be detected on human cancer cells [24]. NKG2D expression is not restricted to NK cells but has also been detected on subsets of T cells, including recently activated CD8 + T cells, γδ T cells and iNKT cells [25]. NKG2D ligands include MHC Class I chain A and B (MICA and MICB), and UL16-binding proteins [4]. Expression of these ligands in a panel of NSCLC cell lines and one patient-derived model was highly heterogeneous [26]. MICA expression was the most consistent, with 4/6 cell lines showing expression above 90%. MICB expression ranged from 1 to 95%, ULBP1 0–6% in 5 cell lines but one line showing expression in 99% of cells. ULBP2 expression ranged between 0% and 98%, and ULBP3 between 11% and 99%. Out of the 6 cell lines studied, H1155 consistently had expression of these NKG2D ligands above 90%, whereas the other cell lines had variable expression of NKG2D ligands [26].

NKG2A is an inhibitory receptor that forms a heterodimer with CD94 and is found on about 50% of peripheral NK cells and on a subset of CD8 + T cells [27]. In several tumor types such as melanoma, breast, and liver cancers, NKG2A was enriched in the CD56bright NK population [27]. HLA-E binding to NKG2A/CD94 signals through immune receptor tyrosine-based inhibitory motifs (ITIM) that result in NK and T cell inactivation, and physiologically this prevents development of autoimmune diseases [27]. CRISPR-knockout of NKG2A in primary human NK cells enhanced killing of a B lymphoblastoid cell line, however also decreased NK cell expansion [28]. They surmised that through inhibiting proliferative activity of NK cells and activation-induced cell death, and thus while NKG2A does serve as a checkpoint, a downstream consequence could be preserving the expansion ability of NK cells [28]. NK cells in many solid tumors including breast, lung, and liver cancers show increased expression of NKG2A, and likewise HLA-E is frequently increased in melanoma and cancers of the lung, kidney, liver, prostate, and colon [27, 29].

The leukocyte Ig-like receptor (LIR, or LILR) family is expressed on multiple lymphocytes including NK, T, and B cells, and antigen-presenting cells including macrophages and dendritic cells [30]. This family contains receptors that activate (LILRA1-6) and inhibit (LILRB1-5) leukocyte function. Similar to NKG2A, inhibitory signals are transduced through ITIMs upon ligation by HLA class I molecules [30]. LILRB1 was identified in circulating CD56dim NK cells from patients with prostate cancer and multiple myeloma, and LILRB1 expression was upregulated compared to healthy donors [31]. HLA-G expression in melanoma cells signaling through ILT2 (LILRB1) prevented NK cell cytolysis in vitro [32]. In cancer, NK cells can also be induced to express inhibitory checkpoints such as PD-1, TIGIT, TIM-3, and LAG3. These are discussed in more detail in the section “Immune checkpoints on NK cells”. Taken together, NK cell cytotoxic function is tightly regulated by the balanced ligation of inhibitory and activating receptors, which can be dysregulated in solid tumors preventing NK cell cytotoxicity.

### NK cell (dys)function in anti-cancer immunity and opportunities for therapeutic interventions

#### Association of NK cell tumor infiltration with clinical outcome

NK cells have been shown to potently eliminate tumors in various orthotopic and spontaneous models of cancer in mice (including melanoma, lymphoma, sarcoma, and colon cancer), highlighting the potency of NK cells in anti-tumor immunity [33–35]. Several studies in human cancer patients found that NK cell infiltration and NK cell activity predicts favorable clinical outcome in a number of malignancies, including melanoma, gastric cancer, and head and neck cancer [36–38]. The positive prognostic effect of NK cells in metastatic melanoma was further enhanced with concomitant expression of IL15 [37].

NK cell signatures also affect prognosis in lung cancer. A seven-gene signature of NK cells was predictive of response to immunotherapy and favorable prognosis in lung adenocarcinoma [39]. Another study identified a FLT3 (FMS-related tyrosine kinase induced in dendritic cells) axis in the NSCLC Cancer Genome Atlas (TCGA) database. High FLT3 expression correlated with a gene signature indicative of high immune infiltration (particularly of NK cells and DCs), increased expression of cGAS-STING effectors, and increased disease-free survival in both squamous and adenocarcinoma histologies [40]. Presence of NK cells in primary lung tumors have been correlated with improved patient survival in NSCLC [41]. Furthermore, depletion of NK cells reduced tumor clearance and enhanced metastasis in several murine models of lung cancer [7, 42].

Meta-analysis of TCGA datasets showed that several NK cell receptors are associated with increased overall survival across different tumor types, suggesting that NK cells are indeed beneficial for tumor control [43]. However, there was a strong correlation between CD8 + T cell and NK cell infiltration, indicating that T cell immunity, NK cell immunity, or a combination of both may be responsible for improved overall survival in these cases. Moreover, NK cells stimulated recruitment of cDC1 into melanoma tumors, promoting cancer immune control, and highlighting the immunomodulatory role of NK cells [41]. Taken together, there is strong evidence that NK cells promote anti-tumor immunity in multiple malignancies, which may be the result of direct elimination of tumor cells by NK cells, improved T cell responses, and/or immunomodulatory function of NK cells within tumor microenvironments (TME).

Certain subsets of NK cells have been associated with favorable responses. The CD57 + tumor infiltrating NK subset (detected by IHC) was correlated with survival after curative intent resection in squamous cell lung carcinoma [44]. Recent studies using multiparameter flow cytometry of human tissues showed that the proportion of NK cells within primary lung tumors were lower than in adjacent normal lung. NK cells composed about 4.5% in primary lung tumors, compared to 47% of T cells [45]. Interestingly, the CD16-negative NK population was similar between tumor and non-tumor sections, whereas CD16 + NK cells were decreased in tumor sections relative to distal lung [45]. Another study of NK cells in lung cancer demonstrated that the majority of tumor-infiltrating NKs lacked CD16 expression, and observed that the CD69 + tissue-resident NK cells (trNK) subset was enriched in the tumor center vs. distal lung in both adenocarcinoma and squamous lung histologies [46]. Interestingly, expression of checkpoint inhibitors TIM3 and TIGIT (but not PD-1) on trNK cells increased closer to the tumor center. trNK’s enriched in tumor centers had greater enrichment of CXCR6, lower expression of CCR2, and expressed Granzyme A and Granzyme B, but not perforin [46]. In another study including 30 NSCLC patients, NK cells ranged between 1.7% and 34.4% of tumor infiltrating lymphocytes, most being CD56^dim^, and associated with downregulation of activating receptors such as CD16 and NKG2D [47]. Future studies leveraging spatial transcriptomics and/or multiplexed immunofluorescence would be valuable to better understand the topography of NSCLC tumors including stromal and peri-vascular niches within the tumor.

Understanding how NK cells may evolve over the course of NSCLC treatment could lead to discovery of new vulnerabilities. Only a few studies have described how NSCLC therapies affect NK cells. One study evaluated NK cell precursors in peripheral blood at baseline and after one cycle of standard treatment in a cohort of 18 patients with metastatic lung cancer [48]. 10 patients received chemo-immunotherapy, 7 patients received immunotherapy alone, and 1 patient received Osimertinib (indicating presence of an activating EGFR mutation, of note these patients are not standardly treated with immunotherapy). They identified a 5-fold increase in Lin-CD34 + DNAM-1^bright^ NK cell precursors, but not total circulating CD34 + precursor cells after just one cycle, suggesting a selective induction mobilization of inflammatory precursors. They also showed that the precursor cells in the primary tissue preferentially expressed CXCR4 compared to peripheral blood. Another study compared the effect of two standard NSCLC drugs gemcitabine (chemotherapy), and gefitinib (an EGFR TKI), on NKG2D ligand expression and NK cell activity [49]. The NKG2D ligands examined in this study included MHC Class I chain related genes A and B (MICA and MICB) and UL16-binding proteins (ULBPs 2,5, and 6). Gemcitabine treatment upregulated NKG2D ligands and concomitantly promoted NK cell anti-tumor activity. Conversely, gefitinib downregulated NKG2D expression and decreased NK-mediated cell lysis. Not all cytotoxic agents, including docetaxel, pemetrexed, or vinorelbine, were able to modulate the NKG2D axis. This suggests that some but not all chemotherapies can augment NK cell anti-tumor activity. Taken together, these data suggest that understanding the kinetics of NK cell activity after standard chemotherapies could identify optimal sequencing of NK cell augmentation therapies in combination approaches, and that each chemotherapy (or targeted therapy) may have distinct effects on NK cells.

#### NK cells in immunosuppressive tumor microenvironments

**Fig. 1:**
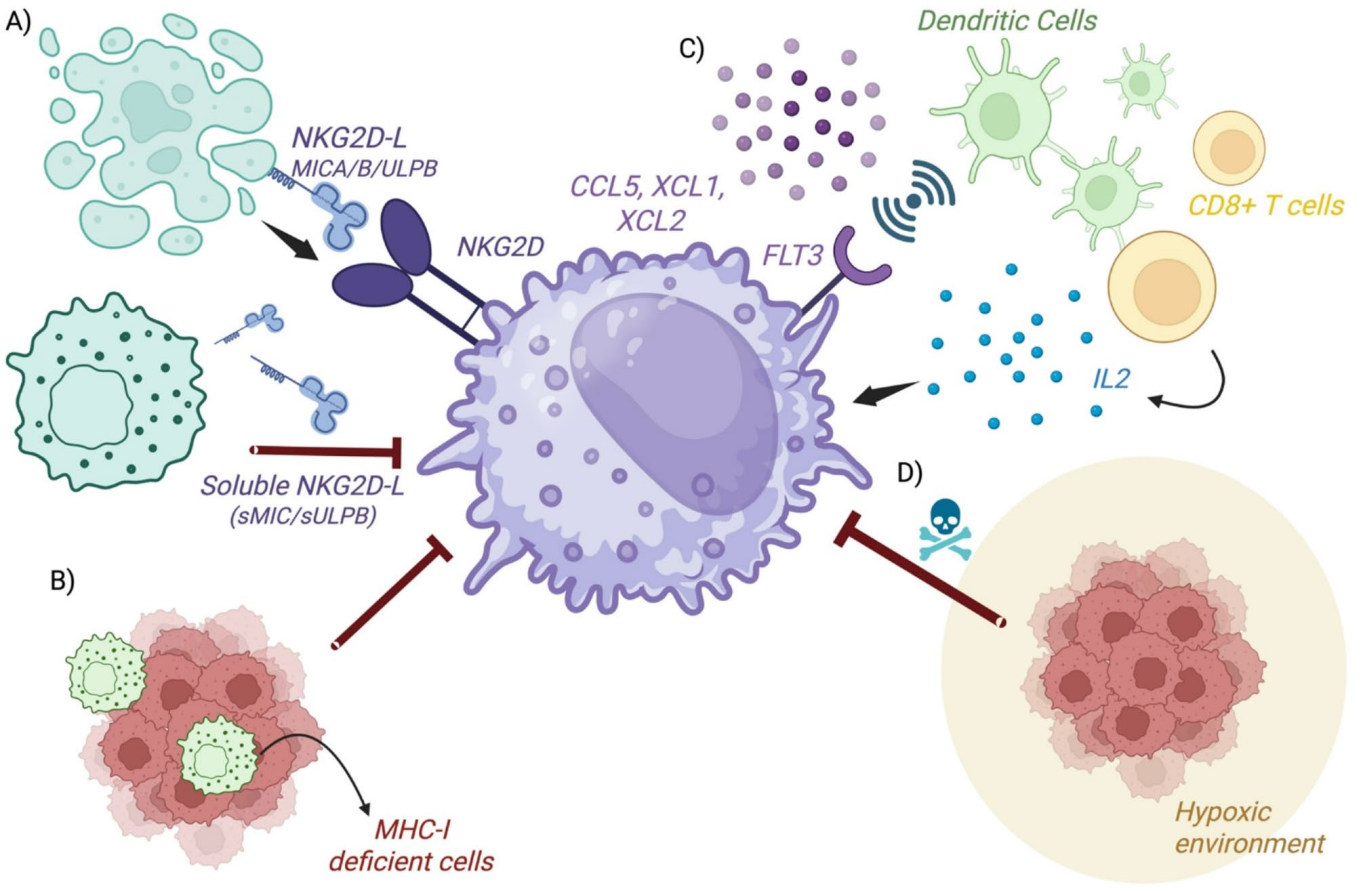
NK cells and tumor interactions^a.^ (**A**) NK cells recognize and target tumor cells primarily though activating receptors like NKG2D. NKG2D ligands (NKGD2-L) are often expressed on tumor cells, however, tumor cells can avoid NK detection by cleaving NKG2D-L, including MICA, MICB, and ULPB [139, 142]. (**B**) A subset of NSCLC harbor mutations in MHC-I antigen processing and presentation machinery, leading to MHC-I loss and immune evasion [33, 148–150]. However, NK cells can become tolerant even when as little as 10% of tumor cells are MHC-I deficient [157]. (**C**) NK cells have regulatory functions outside of non-cytotoxic functions of NK cells at the tumor site. NK cells produce chemokines CCL5, XCL1, and XCL2 to recruit dendritic cells to the tumor site. NK cells also produce FLT3L which facilitates differentiation and survival of dendritic cells [40]. This recruitment of dendritic cells amplifies induction of the CD8 T cell response. IL-2, which is produced and secreted by T cells activates NK cells [245, 246]. (**D**) Many solid tumors exist in hypoxic environments [94]. There are several mechanisms in which hypoxia can impede NK cell activity, including mitochondrial fragmentation in NK cells, transcriptional reprogramming of NK cells, and downregulation of activating NK receptors. Hypoxia also activates the HIF1α axis, which inhibits IL-18 signaling in NK cells [66, 67, 95, 96].

NK cells isolated from lung tumors exhibited limited cytotoxic activity [47, 50], indicating that NK cell function can be dampened within tumor microenvironments. The following sections discuss potential mechanisms of suppression within TMEs, with particular note to bone and brain metastases given the high frequency and devastating clinical complications of these metastatic sites in lung cancer. Figure 1 depicts common mechanisms of NK cell suppression by tumors. 

To dissect potential mechanisms of NK cell dysfunction, Platonova, et al., examined NK cell receptor expression in primary human NSCLC samples compared to matched peripheral blood NK cells and non-tumoral distant tissue [47]. Overall, activating receptors NKp30, NKp80, CD16, NKG2D, and DNAM-1 was significantly downregulated compared to matched peripheral blood NK cells or NK cells from healthy donors. In contrast, NK cells in these lung tumors expressed higher levels of the inhibitory receptor NKG2A [47]. In vitro co-cultures of human peripheral blood NK cells with the A549 NSCLC cell line resulted in downregulation of the activating receptors NKp30, NKp80, and DNAM-1 in the NK cells [47]. Similarly, the inhibitory checkpoint NKG2A was upregulated in an in vivo metastasis model of NSCLC [51]. Evidence that tumor cells downregulate cytolytic functions of NK cells by tumors was demonstrated in vivo, in a syngeneic model of colon adenocarcinoma that employed temporal photoconversion of NK cells to track tumor-infiltrating NK cells [52]. Dean, et al., showed that peripheral NK cells infiltrating into tumors became converted into a tissue-resident phenotype lacking cytolytic function [52]. These dysfunctional NK cells expressed CD49a, increased expression of inhibitory checkpoints, and exhibited decreased chemokine and cytokine production. However their function was improved by administration of an IL-15:IL-15Ra complex [52], suggesting that this NK cell constraint has the potential to be reversed.

Similar to the adaptive anti-tumor T cell immunity, tumor-infiltrating NK cells are susceptible to immune modulating factors within the tumor microenvironment (Reviewed in [53, 54]. For example, extracellular adenosine and prostaglandins suppress anti-tumor NK cell responses [55, 56]. In mouse models of melanoma and sarcoma, tumors produced suppressive cytokines such as TGF-beta that modulated NK cells to a ILC1 phenotype with decreased anti-tumor function [57, 58]. Corresponding with this observation, TGF-beta downregulates several activating receptors in NK cells in the context of NSCLC and breast cancer [47, 59, 60], and plasma levels of TGF-beta inversely correlated with NKG2D expression in patients with lung or colon cancer [61]. Inhibition of TGF-beta using small molecule inhibitors or neutralizing antibodies reversed NK suppression in NSCLC and breast cancer in vitro [47, 59, 60], in ovarian cancer in vivo [62], and increased in vivo NK cell frequency in oral squamous cell carcinoma [63]. Engineered NK cells resistant to TGF-beta (via Smad4 knockout or expression of a dominant negative TGF-beta receptor) led to improved anti-tumor activity against breast cancer, colon cancer, and neuroblastoma [64, 65].

Many solid tumors are hypoxic, which causes mitochondrial fragmentation in tumor infiltrating NK cells, resulting in decreased NK cell function [66]. Furthermore, hypoxia signals through HIF1α to inhibit IL18-signaling in NK cells, reducing NK cell anti-tumor activity [67]. In murine breast cancer models, senescent cancer-associated fibroblasts secreting extracellular matrix dampened NK cell activity [68]. In melanoma, tumor infiltrating NK cells expressed higher levels of the KLRC1 gene (encoding the inhibitory receptor NKG2A) than NK cells in peripheral blood [69]. Thus, NK cell function can be suppressed within TMEs through several mechanisms.

Single cell RNA sequencing (scRNAseq) of early-stage lung cancers after tumor resection showed imbalances in immune cell subsets including NK cells, T cells, and myeloid cells even in stage I NSCLC, suggesting early changes in tumor microenvironment [70]. Of note, 4 out of the 6 tumors analyzed in this manuscript harbored an EGFR activating mutation (3 L858R and 1 exon19 deletion) [70]. EGFR-mutated NSCLC has very limited response to current checkpoint inhibitors [2, 71] and so studies of the immune landscape in cancers with these driver mutations are crucial to understand mechanisms underlying the lack of response to immunotherapy. scRNAseq of 44 patients with NSCLC who had not undergone any systemic therapies reveal several insights into NK cell populations in different sites of disease, including lung, pleural effusions, lymph node, and brain [72]. Number and relative proportion of NK cells declined in NSCLC at all sites compared to normal lung tissues. NK cells were detected in all sites, and had comparable gene expression profiles, except that expression of FCGR3A was suppressed in pleural effusions and somewhat in brain compared to other sites [72]. A similar depletion of NK cells in NSCLC tumors compared to normal lung was also identified by Leader, et al., using CITE-seq analysis [73]. De Zuani, et al., employed combined scRNAseq from 25 patients and spatial transcriptomics from 5 patients, evaluating tumor and adjacent normal lung (background) [74]. Cytotoxic NK cells were decreased in tumor vs. background. The pattern of expression of checkpoint molecules on NK cells appeared to be similar in both squamous and adenocarcinoma histologies of lung cancer, and NK cells in both groups were particularly enriched in CD96, a potential immune checkpoint receptor [75]. In another scRNAseq analysis of NSCLC tumors, expression of the matricellular protein periostin expressed by cancer-associated fibroblasts was inversely correlated with cytotoxic T cells and NK cells, suggesting an additional mechanism of immunosuppression [76].

#### NK cells in bone metastases

Bone is one of the most common sites for metastasis in NSCLC, with approximately 35–60% of NSCLC patients developing bone metastases (BoM) as the disease progresses [77]. The median overall 5-year survival rate from BoM patients is 4–6% [78]. Furthermore, about 46% of BoM patients experience skeletal related events (SREs) that significant impair quality of life and can be life-threatening, including pathologic fractures, spinal cord compression, pain, and hypercalcemia [79]. A retrospective analysis of patients with metastatic NSCLC receiving the anti-PD1 checkpoint inhibitor nivolumab showed that presence of BoM decreased response rates and overall survival compared to patients without BoM [80]. Focused treatments such as ablative radiotherapy, surgery, and bone modifying agents (denosumab and zoledronic acid) can help palliate symptoms and reduce SREs but effects on survival are only modest [81, 82]. Bone is an extremely complex organ with multiple sub-niches such as endosteal, perivascular, and hematopoietic stem niches, containing multiple cell types that can each impact NK cell phenotypes [83].

Gillespie, et al., performed a multi-institution Phase 2 randomized controlled trial comparing prophylactic radiotherapy to sites of asymptomatic, high-risk BoM plus standard of care systemic therapy versus systemic therapy alone [84]. These lesions were designated high-risk for pathologic fracture based on size of the BoM, location in specific weight-bearing bones, and degree of cortical destruction. This study enrolled patients with multiple solid tumors including prostate and breast cancer, yet the NSCLC cohort was the most predominant, indicating the predilection for high-risk BoM in lung cancer. The primary endpoint was rate of SREs and secondary endpoint was survival. As expected, the rate of SREs significantly decreased in the intervention group. Interestingly, survival rates were also significantly improved with radiotherapy [84]. Despite these advances, bone metastases remain a key driver of morbidity, mortality, and decreased quality of life [77, 79, 81].

BoM have been historically difficult to study as bone is a challenging site to biopsy, and bone biopsies frequently require decalcification for routine clinical histological analysis. Kfoury, et al., (part of the Boston Bone Metastases Consortium) designed a translational trial with dedicated procurement of fresh bone metastasis tissue and liquid bone marrow from nearby bone involved with tumor and distal bone within the surgical field (not involved with tumor) [85]. Tissue samples came from patients with prostate cancer, NSCLC, and melanoma. As a benign comparator group, they obtained bone marrow from patients during hip replacement surgery and performed multiparameter flow cytometry and single cell RNAseq in these samples. NK cells were detected in all samples including benign, tumor, and distal or involved bone marrow, and very low in benign bone marrow. NK cells were highly enriched in distal and involved bone marrow samples, but not in tumor BoM, suggesting that NK cells can be recruited to peri-tumoral bone but excluded from the BoM tumor itself [85]. A murine model of Lewis lung carcinoma (LLC) cells selected for high CX3CL1 (also known as fractalkine or FKN) expression showed increased NK cell infiltration and decreased tumor growth in bone metastases [86]. This study also identified enrichment of inflammatory monocytes and B cells, and there was no difference in CD4 + and CD8 + T cell infiltration between the FKNlo and FNKhi tumors [86]. However, fractalkine has multiple functions [87], some that promote tumor metastasis via enhanced NSCLC transendothelial migration [88], underscoring the importance of accounting for tumor genomics and microenvironmental contexts including specific subtypes of key immune cells including NK cells.

Another study included 10 patients with bone metastases from NSCLC, 6 from patients harboring actionable genomic alterations (driver-positive) and 4 driver-negative [89]. A separate cohort of primary lung tumors were used as a comparator. In the bone metastases from driver-positive tumors, a gene signature of activated NK cells was enriched compared to primary lung tumors. Two patients in this cohort received third-line checkpoint inhibitor therapy after standard targeted therapy (tyrosine kinase inhibitors) and chemotherapy. The one patient who exhibited a durable response to nivolumab (stable disease as best response, duration of response 29 months) had higher expression of activated NK cells compared to the patient who had no response to nivolumab (progressive disease at 2.5 months). However, these data must be interpreted with caution due to the small cohort size [89]. Most driver-positive NSCLC (such as those harboring EGFR or ALK alterations) typically do not respond well to standard PD-1/PD-L1 checkpoint inhibitors and are instead treated with target-specific tyrosine kinase inhibitors [2, 71]. The presence of activated NK cells in these bone metastases -raises the hypothesis that NK cell-augmenting therapies may be a promising avenue to treat bone metastases in this subgroup of NSCLC. However, NK cell subsets should be studied in larger cohorts of NSCLC patient samples including those with EGFR and ALK alterations, and NK cell therapies should be tested in pre-clinical models of these specific genomic subtypes of NSCLC.

NK cells controlled bone metastases in models of bone-metastatic breast and prostate cancers [90, 91], and utilizing NK cells to combat metastatic disease is an attractive prospect. NK cells mature and differentiate within the bone marrow, meaning this niche is rich with NK precursors at all developmental stages, while mature NK cells primarily circulate in the peripheral blood (PB-NK) or take residence in other lymphoid/non-lymphoid organs [92]. There are heterogeneous populations of NK cells in bone, including a bone marrow resident NK population that has low cytolytic activity [93]. Thus, there is a great need to further define the functional interactions of NK cells within the BoM TME, as there are both activating and inhibitory signals for NK cells in the bone.

There are many factors within the BoM TME that suppress NK cell function, including soluble factors such as TGF-beta and IDO secreted by tumor cells and immune suppressor cells, and microenvironmental effects such as hypoxia [94]. Hypoxia itself reprograms NK cells and results in downregulation of NK cell activating receptors and limits cytotoxic activity [95, 96]. The RANK/RANKL axis, which promotes osteoclast differentiation and bone remodeling, also acts as a modulator of metastases through angiogenesis and facilitating the migration of cancer cells [97]. NK cells express the RANK receptor, and in the context of acute myeloid leukemia, RANK/RANKL signaling has been shown to inhibit NK cell anti-tumor activity by the release of immunomodulatory factors including TNF, IL-6, IL-8, and IL-10 [98]. The administration of denosumab (a RANKL-neutralizing antibody) partially rescues NK cell killing against solid tumors [99]. Of note, denosumab is already FDA-approved for prevention of SREs in patients with bone metastases [82, 100–102], thus this pathway has high clinical relevance.

**Fig. 2:**
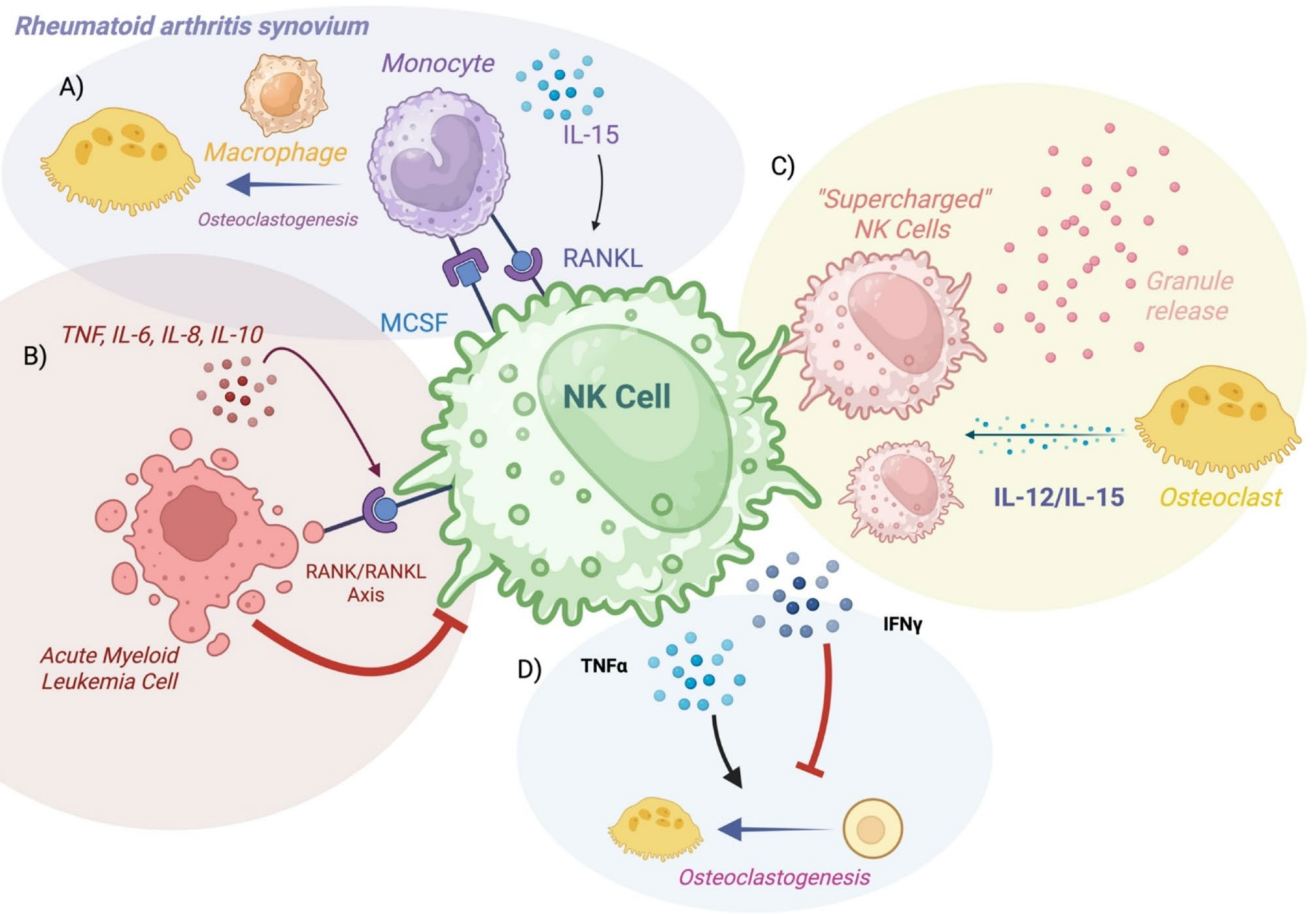
NK cells in bone microenvironments^b^ (**A**) NK cells in the rheumatoid arthritis synovium facilitate cellular differentiation from monocytes to osteoclasts. RA synovium NK cells express both RANK-L and M-CSF, which promotes osteoclastogenesis [105]. Expression of these molecules on NK cells is upregulated by IL-15, an abundant cytokine in the RA synovium. (**B**) Secreted factors produced by acute myeloid leukemia cells induce the expression of the RANK receptor on NK cells. When NK RANK interacts with RANKL on target cells, NK cells display impaired antileukemic activity [98]. (**C**) Osteoclasts have been shown to induce expansion and increase the antitumor functions of NK cells. OCs secrete IL-12 and IL-15, important activators of NK cells. NK cells expanded in the presence of OCs exhibit increase granule release and increased secretion of TNF-α and TRAIL [104, 247, 248]. (**D**) Secreted factors produced by NK cells can influence the differentiation of osteoclasts. IFN-γ has been shown to inhibit osteoclastogenesis in vitro, whereas TNF-α has been shown to increase RANK-L expression on macrophages which helps drive osteoclastogenesis [106–108]

The interaction between NK cells and osteoclasts is complex and varied. Osteoclasts secrete NK stimulating cytokines like IL-12, IL-15, and IL-18 [94], and are able to activate and expand NK cells in vitro [103]. Osteoclasts derived from NK-injected tumor bearing humanized mice were able to facilitate NK cell killing of oral tumor growth [104]. NK cells could also promote osteoclast activity, as shown by a study of rheumatoid-arthritis-associated synovium [105]. NK cells in this environment have been shown to express both RANKL and M-CSF, two key ligands that promote osteoclast differentiation. When synovial-derived NK cells were co-cultured with monocytes, they induced osteoclast differentiation [105]. The presence of TNF-α can increase RANKL expression on macrophages, facilitating osteoclastogenesis [106, 107]. Conversely, the secretion of IFN- γ by NK cells and Th1 lymphocytes has been shown to inhibit osteoclastogenesis in vitro [108]. Osteoclasts express many ligands for NK cell activating and inhibitory receptors such as MHC-I, MIC-A/B, ULBP-1-6, and MHC class I-like ligands [103, 109]. It is important to note that the bone TME is highly complex with multiple cell types [83], and thus NK cell subtype and function may differ depending on the presence or absence of other cell types and disease context (i.e. cancer vs. rheumatoid arthritis). The schematic in Figure 2 highlights key pathways employed in cellular crosstalk in NK cells in bone metastases. 

#### NK cells in brain metastases

Brain metastases (BrM) are a common complication in patients with advanced NSCLC, with approximately 40% of patients developing brain metastases during disease progression [110, 111]. BrM are indicative of poor prognosis, with treated patients displaying a median survival time of 15 months [110, 111]. BrM are believed to originate from hematogenous dissemination of circulating tumor cells present in the brain microvasculature [111]. Activating mutations present in the epidermal growth factor receptor (*EGFR*) and anaplastic lymphoma kinase (*ALK*) are thought to increase patient susceptibility to developing BrM [112, 113]. Again, these genomic alteration-driven NSCLCs demonstrate significantly decreased response to immunotherapy [2, 71], with the exception of individual case reports [89].

The central nervous system (CNS) has been defined as an immune-privileged site; however, experiments have demonstrated that NK cells in mice (comparable to CD56^bright^ cells in humans) can cross the blood brain barrier, gaining access to the CNS [114]. CD3^−^CD56^+^ NK cells constitute 2.5% of infiltrating leukocytes in the brain TME, with 60% of this population expressing the NKG2D receptor [114]. NK cells are recruited to the brain via the CX3CL1 chemokine, which acts on the CX_3_CR1 receptor of NK cells. Glioma cells possessing the isocitrate dehydrogenase (IDH1-R132) mutation produce higher levels of CX3CL1, leading to higher infiltration of NK cells [115]. Several computational-based studies show strong correlations between activated NK cells and improved survival in glioblastoma patients [116–118]. Although these results were identified in a primary CNS tumor, they underscore the importance of NK cells in tumor eradication and demonstrate that NK cells can be recruited to brain microenvironments.

**Fig. 3:**
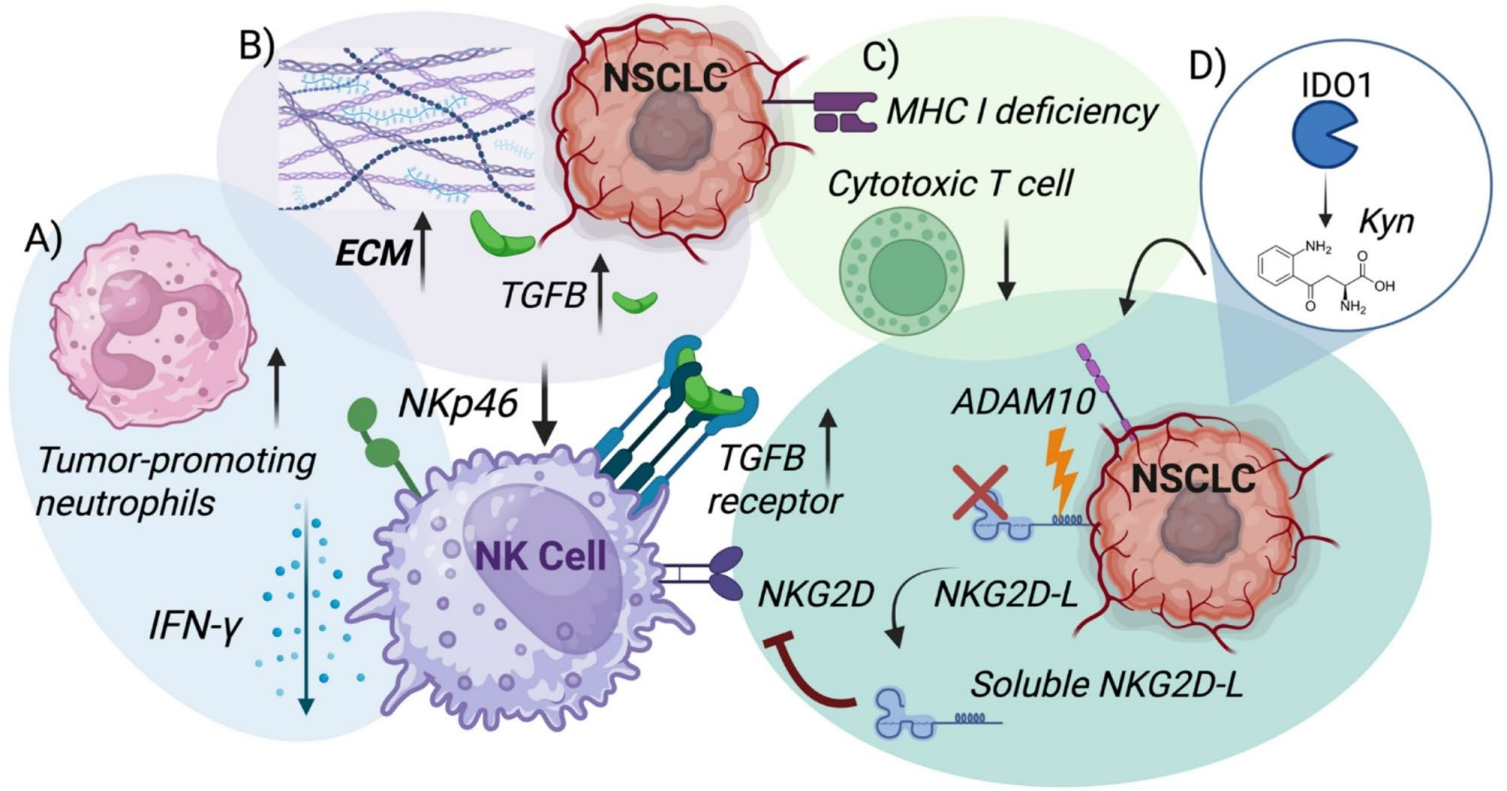
An immunosuppressive brain metastasis microenvironment^c.^ (**A**) Lung cancer brain metastases are enriched in tumor-promoting neutrophils [119, 120, 123]. While neutrophils can differentially modulate NK cell activity depending on different microenvironmental contexts, in tumors, neutrophils can downregulate the activating receptor NKp46 on NK cells and dampen interferon-gamma release [124]. (**B**) Lung cancer brain metastases are enriched in TGF-beta and extracellular matrix (ECM) proteins [119, 120]. ECM downregulates cytotoxic programs in NK cells, and this is associated with upregulation of the TGF-beta receptor on NK cells in tissues, favoring transition to a tissue-resident NK subtype that has decreased cytotoxic activity [249]. (**C**) Brain metastatic NSCLC frequently shows downregulation of MHC class I molecules, a mechanism of T cell immuno-evasion [119, 120]. Although NK cells are licensed to eradicate MHC deficient tumor cells, the inhibitory effects of ECM and TGF-beta on NK cell cytotoxicity (as discussed in 3B) prevent NK cells from eliminating these MHC deficient tumors. (**D**) Overexpression of indoleamine 2,3-dioxygenase 1 (IDO1) (an enzyme that converts tryptophan to kynurenine (Kyn)) in brain metastases can regulate the expression of the ADAM10 receptor. IDO1 overactivity and subsequent over expression of ADAM10 results in release of NKG2D ligands from cell membranes (NKG2DL, lightning bolt indicates cleavage from membrane) on tumor cells to generate soluble NKG2D ligand, impairing NK cell cytotoxicity [122]. Created in BioRender. Sethakorn, N. (2025) https://BioRender.com/74mvyml. Created in BioRender. Sethakorn, N. (2025) https://BioRender.com/d8rsuzj^b.^ Created in BioRender. Sethakorn, N. (2025) https://BioRender.com/e71bmt0^c^

A single cell atlas of metastatic brain tumors (including 3 patients with NSCLC) showed downregulation of MHC class I molecules in the tumor cells [119]. Spatial transcriptomic analysis of primary lung tumor and brain metastases in NSCLC showed decreased enrichment of NK cells in brain vs. lung, both in the immune regions and tumor regions [120]. Single cell analyses of BrM and primary lung tumors showed similar trends in paucity of NK cells, depletion of activated and effector T cells, and enrichment of Tregs and naïve T cells [121]. They also identified chromosomal instability (CIN) high features and cGAS + micronuclei in BrM, and hypothesized that this led to tonic cGAS-STING signaling that induces immunosuppression that affects multiple immune cell types [121]. There may also be tumor-specific differences in BrM, for example, NKG2D ligand expression was inversely correlated to the immunosuppressive factor IDO1 expression in BrM [122]. Neutrophils have been shown to be enriched in lung cancer BrM [45, 119, 120, 123], and while neutrophil function is diverse and context-dependent, they can promote tumor progression [124]. Neutrophils are able to downregulate cytotoxic receptors and decrease IFN-gamma secretion by NK cells, reflecting some potential tumor-permissive mechanisms [124]. Figure 3 shows key pathways mediating NK cell suppression in brain metastases. 

Overall, CNS tumors and BrMs still often display low levels of tumor infiltrating lymphocytes, suggesting the CNS tumor microenvironment exerts a variety of immunosuppressive mechanisms contributing to tumor resistance [125]. Despite these observations, immunotherapy-based approaches including anti-PD1/PD-L1 and anti-CTLA4 have demonstrated intracranial activity in melanoma and NSCLC [126]. Furthermore, the bispecific T cell engager tarlatamab has also shown promising intracranial control with shrinkage of at least 30% in intracranial lesions observed in 62.5% of patients with refractory small cell lung cancer (SCLC), although the CNS lesions did have prior radiotherapy [127]. Tarlatamab is now FDA-approved in extensive stage, treatment-refractory SCLC [128]. These data indicate that while the brain TME exhibits multiple immunosuppressive mechanisms, there is opportunity for rationally-designed therapies to induce anti-tumor immune responses in BrM.

#### Pro and anti-tumor activity of NKG2D and its ligands

Overexpression of NKG2D-L on several mouse tumors leads to increased NK and CD8 + T cell-dependent tumor control [129]. Some tumors evade recognition by NKG2D by cleavage of NKG2D-L, including MICA [129–131]. Fang, et al., demonstrated that NSCLC cells upregulated IDO1, leading to increased ADAM10 expression and subsequent ADAM10-induced shedding of NKG2D ligands [122]. Inhibition or deficiency of IDO1 enhanced NK cell anti-tumor activity against NSCLC in vitro, and a combination of an IDO1 inhibitor with NK cells was more efficacious in tumor killing than either alone [122]. Several strategies are being developed to prevent the negative effects of soluble NKG2D-L to improve recognition by NK cells, including blocking the cleavage of MICA and MICB [132]. Despite its role in mediating potent anti-tumor responses, NKG2D has also been implicated in pro-tumorigenic roles under certain conditions. For example, NKG2D-deficient mice exhibit enhanced tumor control in models of prostate carcinoma, inflammation-driven hepatocellular carcinoma, B16 melanoma, and radiation-induced lymphoma. Conversely, these mice show accelerated onset of Myc-driven lymphomas, highlighting a context-dependent role for NKG2D in anti-tumor activity [133–135]. Increased tumor control was associated (in part) with increased function of the activation receptor NKp46 [134]. Additionally, endothelial cells can express NKG2D-L and thereby desensitize antitumor NK cell responses [136]. Moreover, increased *KLRK1* (encoding NKG2D) expression was associated with decreased overall survival in colorectal cancer, suggesting that NKG2D reduces tumor control in certain human tumors [137]. Thus, NKG2D can mediate both anti- and pro-tumorigenic effects in vivo.

Depending on cellular context, membrane-bound NKG2D ligands and soluble NKG2D ligands can exert differential effects on NK cells, with soluble NKG2D ligands suppressing NK cell function in lung cancer [3]. One example is MIC, which can be cleaved to generate soluble MIC, or sMIC [3]. Of note, the effect of these NKG2D ligands depended on the model system and whether they were membrane-bound or soluble, highlighting effects that were highly context-specific [138]. Dhar, et al., showed that membrane-bound MIC activated NKG2D to potentiate anti-tumor NK cell responses in the TRAMP/MIC model of prostate cancer, however sMIC activated signalosome activity in NK cells leading to NK cell suppression [139]. In patients with prostate cancer, peripheral NK cells were reduced and sMIC levels were high compared to healthy control individuals [140]. Serritella, et al., demonstrated that Lewis lung carcinoma (LLC) with overexpressed sMIC significantly had increased tumor volume and growth rate in a syngeneic mouse model [138]. The percentage of NK cells and interferon-gamma-expressing NK cells within tumor-infiltrating leukocytes were also significantly decreased in sMIC expressing Lewis lung carcinoma implants [138]. Lung tumors with high expression of MIC correlated with decreased NKG2D expression on TILs [130].

An antibody designed to neutralize sMIC and clear sMIC levels without blocking NKG2D activation restored NK cell anti-tumor activity in mouse models of melanoma and prostate cancer [141, 142]. Mice with high sMIC levels responded poorly to anti-CTLA4 blockade, which was reversed with the sMIC antibody in a MIC transgenic spontaneous TRAMP model of prostate cancer [143]. Notably, the sMIC neutralizing antibody also enhanced tumor response to PD-L1 blockade in the same model of prostate cancer [144]. This approach may be translatable to other solid tumors that have detectable circulating sMIC, including melanoma in which sMIC expression correlated with reduced overall survival in response to immunotherapy but not chemotherapy or BRAF-targeting therapy [145]. Soluble MICB levels correlated with increasing stage and risk of metastasis in multiple solid tumors including lung, breast, gastrointestinal, genitourinary, and gynecologic cancers [146].

Another NKG2D ligand, ULBP proteins, can also be cleaved to generate soluble versions [3]. sULBP-1 was also correlated with reduced survival in patients with melanoma treated with immunotherapy [145]. sULBP-2 was found at high levels in serum from patients with NSCLC and particularly high in those with squamous histology [147]. Notably, high sULBP2 expression correlated with poor overall survival (10.8 vs. 16.8 months) in a cohort of patients with stage III/IV NSCLC, and this association was identified in univariate as well as multivariate analysis including stage and histology [147]. These findings also highlight the importance of examining protein biomarkers, given that these associations may not have been identified on transcriptomic and genomic analyses. These data also highlight the importance of examining protein-protein network signatures, for example, receptor-ligand pair interactions and or substrate/enzyme interactions relevant to cleavage of membrane-bound NK cell ligands. Based on these studies, soluble NKG2D ligands may be a new promising liquid biomarker in lung cancer, both as a prognostic level as well as predictive biomarker to standard checkpoint inhibitors and potentially novel sNKG2D ligand targeting approaches.

### NK cell dysfunction in MHC-I deficient tumors

To evade being recognized by CD8 + T cells, some tumors lose MHC-I expression thereby becoming potential targets for NK cells, however this NK cell surveillance is not always successful as patients present with mutations in the MHC-I antigen processing and presentation machinery [33, 148–150]. This type of acquired resistance is gaining interest in the context of immune checkpoint therapy as well [151]. The failure of NK cells to mediate effective immunity against MHC-I deficient tumors may be explained in part by NK cell licensing or education. NK cells acquire functional competence through recognition of MHC-I by their cognate inhibitory receptors Ly49s in the mouse and KIRs in humans, a process termed licensing [152–154]. NK cells can become “unlicensed” or tolerant in MHC-I deficient environments [155, 156]. Unclear is whether the process of “unlicensing” utilizes the same molecular mechanism as licensing, especially within tumor microenvironments, therefore we will use the term tolerance for the purpose of this review. NK cell tolerance occurs even when a minority, as low as 10%, of the cells in the microenvironment are MHC-I deficient [157]. A KIR2D-specific antibody to harness NK cell missing-self responses failed in phase 2 trial [158], which likely can be explained by tolerance in a MHC-I deficient environment that this antibody mimics [159]. This hyporesponsive state can be reversed by inducing a proinflammatory response, such as poly(I: C) injection or virus infection [157, 160]. In experimental MHC-I deficient tumor models NK cell dysfunction can be reversed by cytokines including (engineered) IL-2 and combinations of IL-12 and IL-18 which promotes tumor clearance [161, 162]. Moreover, agents including STING agonists and NKT cell activators can mediate NK cell immunity against MHC-I deficient tumors through type I IFN and IL-21, respectively [163, 164]. To what extent these agents will benefit cancer patients is the subject of ongoing clinical trials [54].

While NK cell dysfunction occurs within the tumor microenvironment of solid tumors, this does not always apply to metastasizing tumors. For example, MHC-I loss is common in primary colorectal tumors, yet colorectal liver metastases are MHC-I positive [165], suggesting that NK cells are unable to eliminate MHC-I deficient tumors in the primary tumor, while NK cells in the circulation are capable to reject these MHC-I deficient tumor cells when they metastasize. Consistent with these observations, mice with specific NK cell proliferation defects are capable to control metastasizing tumors, while they are defective in controlling the same tumors as experimental solid tumors [166]. Thus, the requirements for NK cell-dependent tumor control differs between solid and metastasizing tumors.

### Therapeutic targeting of NK cells

A variety of NK-cell targeting therapies have been tested, ranging from cellular therapy to removal of NK cell checkpoints, and augmenting NK cell activation [7, 29]. Table 1 summarizes past and current clinical trials of NK-cell targeting therapeutics in lung cancer, and Table 2 summarizes clinical trials of NK-cell-based therapies in other solid tumor types. 

#### Targeting NK cells to surface antigens

Besides directly recognizing tumor cells, NK cells can also detect and kill antibody coated tumor cells through a process known as antibody-dependent cytotoxicity (ADCC) [167]. NK cells recognize antibodies primarily through FcγRIII receptor CD16, which can be cleaved off the cell surface by ADAM17 [168]. Several strategies are being developed that bypass complications of CD16 cleavage to improve NK cell ADCC, including NK cell engagers and chimeric antigen receptor (CAR) engineered NK cells [169, 170].

The bispecific T cell engager tarlatamab was approved for treatment of refractory small cell lung cancer, with durable disease control seen in a subset of patients [128]. This success supports the development of other therapeutics such as NK cell engagers that target both a tumor antigen and NK cells, aiming to improve NK cell recognition and function [169, 171]. These engagers under development target both solid and hematological malignancies and several are in clinical trials and include TriKEs and ANKETs. Second generation TriKEs have 3 arms that target CD33, CD16 and have an IL15 moiety that enhances NK cell function, proliferation, and survival which improved tumor control in humanized mouse models [172]. ANKETs target a tumor antigen, CD16, and the activation receptor NKp46 which was effective in a mouse model of lymphoma [173], ANKETs that also target IL-2R are currently under development (https://www.innate-pharma.com/science/our-nk-cell-engager-platform). Thus, therapeutics targeting tumor antigens that have improved NK cell ADCC are promising approaches.

Antibody-based therapies against tumor antigens are expanding in the treatment of solid tumors, including the HER2-targeting antibody trastuzumab first used in the treatment of HER2-positive breast cancer, EGFR-targeting antibody cetuximab approved in head and neck cancers and colorectal cancers [174, 175], and the MET/EGFR bispecific antibody amivantamab approved in NSCLC subtypes [176, 177]. Of note, infiltration of NK cells in breast cancers correlated with response in patients treated with trastuzumab. Mechanistically, CD16 + NK cells produced IFN-gamma and CCL5, which then increased tissue-resident CD8 + T cells [178].

There are also antibody-drug conjugates (ADCs) that consist of an antibody against a tumor antigen conjugated to a cytotoxic chemotherapy payload, and are rapidly being developed against multiple solid tumors and hematologic malignancies [179]. Trastuzumab-based HER2-ADCs are approved in HER2-positive breast cancer, HER2-mutated NSCLC, and HER2-high solid tumors [179–181]. The anti-Trop2 antibody hRS7 was able to induce antibody-dependent cellular cytotoxicity against ovarian and cervical carcinoma cell lines [182–185]. The hRS7 anti-Trop2 antibody is the base for the antibody drug conjugate Sacituzumab-govitecan, which has been approved for treatment in breast cancer, and is under active investigation in phase II-III trials in NSCLC and prostate cancer [179, 186], and examined in combination with immunotherapy [1].

#### Immune checkpoints on NK cells

Immune checkpoint therapy targeting the inhibitory receptors CTLA-4 and PD1 have emerged as an effective therapy for a range of cancers [187]. These immune checkpoints are generally considered to be targeting T cell immunity, but PD1 has been associated with NK cell tumor immunity as well [188], including NSCLC [189]. However, the role of PD1-expression by NK cells has been controversial [43, 190]. While human NK cells have been reported to produce PD1 transcripts in response to dexamethasone in vitro [191], others did not detect PD1 transcripts or evidence of transcription in a PD1 reporter mouse [190, 192]. A potential explanation is that NK cells do not express PD1 transcripts, but can acquire PD1 molecules from tumor cells through trogocytosis [193]. This process required members of the SLAM receptor family to be expressed by the tumor, which is restricted to the hematological compartment under steady state conditions. These observations suggest that PD1 function on NK cells may be restricted to specific malignancies. Other inhibitory checkpoints implicated in NK cell tumor immunity include LAG-3, NKG2A, TIM-3, and TIGIT [194]. LAG-3 is the third immune checkpoint inhibitor that received FDA approval [195]. It is expressed on various immune cells, including NK cells, activated and exhausted T cells, and dendritic cells, and can bind to multiple ligands such as fibrinogen-like protein 1 (FGL1) on tumor cells and MHC-II on antigen-presenting cells [196]. While LAG-3 blockade is associated with improved NK cell responses [197], the impact of LAG-3 expression by NK cells on tumor control is unclear.

NKG2A forms a heterodimer with CD94 and is found on about 50% of the NK cells and on a subset of CD8 + T cells, which is increased after antigen specific stimulation such as vaccination. Inhibiting NKG2A can unleash both T and NK cell-mediated anti-tumor immune responses [198, 199]. The checkpoint inhibitor Monalizumab targets NKG2A and is currently in phase 3 clinical trials [29, 194, 198]. Expression of NKG2A and PD-L1 have been identified on both NK cells and cytotoxic T cells [188, 190] and thus a combination approach with Monalizumab and Durvalumab, a PD-L1 inhibitor, has been explored in multiple trials [200–203]. A phase 1/2 trial of this combination was studied in multiple advanced solid tumors, including NSCLC [203]. Eligible patients had advanced, recurrent, or metastatic solid tumors, had received 1–3 prior lines of therapy, and were immunotherapy naïve. In part 2, there were 20 patients with NSCLC who were treated with Durvalumab 1500 mg every 4 weeks and Monalizumab 750 mg every 2 weeks. 10 patients had a PD-L1 expression level < 25%, 6 patients ≥ 25%, and 4 patients were unknown. Amongst the NSCLC cohort, the objective response rate (ORR) was 10% with a disease control rate of 40% at 16 weeks and 25% at 24 weeks. Median progression free survival was 1.9 months and median overall survival was 8.8 months. In part 2, 20 (14.3%) of patients experienced grade 3–4 treatment related adverse effects, including ALT elevation, anemia, colitis, and hypokalemia. Translational analysis revealed that there was increased peripheral expression levels of the NK activation marker CD38 on CD56^bright^ NK cells and increased levels of CXCL10 and CXCL11. At week 9 of treatment there was increased intratumoral proliferation of CD3^+^Ki67^+^ cells, granzyme B^+^(GZMB^+^) cells, and CD8^+^ cells but no significant increase in intratumoral NKp46^+^ cells [203].

CD73, a 5’ ectonuclease expressed on regulatory T cells and recently identified on regulatory NK cells in lung tumors, represents another novel target [204]. Neo, et al., demonstrated that CD73 + NK cells repressed T cell proliferation and interferon-gamma production via IL-10 production [204]. The first-in-human study of Oleclumab, an anti-CD73 monoclonal antibody, in patients with advanced, treatment-refractory solid tumors showed only modest response rates (less than 10%) in patients with NSCLC, however newer studies looking at combination therapies demonstrate more promising results [205]. The Phase 2 COAST trial studied the role of consolidative Durvalumab alone or in combination with Monalizumab or Oleclumab after the completion of concurrent chemotherapy and radiation for unresectable stage III NSCLC [202]. The confirmed ORR was higher in the Durvalumab plus Monalizumab group (35.5%) and the Durvalumab plus Oleclumab group (30.0%) as compared with the Durvalumab monotherapy group (17.9%). 12-month progression free survival was also improved in the combination groups; Monalizumab 72.7% and Oleclumab 62.6%, as compared with Durvalumab alone, 33.9%. No new or significant safety signals were identified with the combinations [202]. The promising results from this trial prompted the phase 3 PACIFIC-9 trial which started enrollment in February of 2022 and is currently ongoing [200].

Treatment of NSCLC has been advancing to neoadjuvant and perioperative approaches in which chemo-immunotherapy is utilized prior to surgery with the goal of achieving pathologic complete response or major pathologic response. Treatment with immunotherapy with the tumor in place theoretically can induce an individual vaccination like effect with ongoing effective anti-tumor immune surveillance to eliminate micrometastases even after surgical resection [206, 207]. The Phase II NeoCOAST platform study tested novel combinations of durvalumab plus novel immunotherapeutics, and showed safety and feasibility of neoadjuvant durvalumab plus monalizumab in patients with stage IA3-IIIA resectable NSCLC [201]. 84 patients were included across the 4 treatment arms. Major pathologic response rates and pathologic complete response rates respectively were 11.1% and 3.7% with Durvalumab alone and 30% and 10.0% in the Durvalumab plus Monalizumab group. RNA sequencing of pre-treatment and resected tumor samples showed transcriptomic changes after treatment. Gene expression associated with NK cells (*KLRC1*,* GNLY*) and CD8 T cells (*CD8A*,* GZMK*) increased in all treatment arms, especially in the monalizumab and oleculumab combination arms [201].

The results from this trial inspired the NEOCOAST-2 trial, a phase 2 perioperative trial for stage IIA to IIIB NSCLC. During this time, the neoadjuvant therapy space evolved in NSCLC to incorporate approval of standard of care Neoadjuvant/peri-operative chemoimmunotherapy regimens with combinations involving nivolumab, pembrolizumab, or durvalumab [208–211]. Therefore, the NeoCOAST-2 study added chemotherapy to the immunotherapy combinations. NEOCOAST-2 involves Durvalumab plus chemotherapy in combination with novel agents including Monalizumab as neoadjuvant therapy followed by adjuvant immune checkpoint inhibition (ICI). Patients received 4 cycles of neoadjuvant treatment, followed by surgery, and up to 12 months of adjuvant treatment. In the Durvalumab plus Monalizumab group there was a pathologic complete response rate of 26.7% and major pathologic response rate of 53.3% [212].

Four targets have been characterized for TIM-3, which are galectin-9, phosphatidylserine, high mobility group box 1 (HMGB1), and carcinoembryonic antigen-related cell adhesion molecule 1 (CEACAM-1) [213]. TIM-3 has been identified on several immune cell types, including T cells, NK cells, myeloid cells, and B cells [213]. TIM-3 blockade increased NK cell reactivity in the blood from melanoma patients in vitro [214]. Moreover, dual blockade PD1 and TIM-3 suppressed outgrowth of MHC-I deficient tumors in mice [215], suggesting that TIM-3 may play a role on NK cells in vivo but is not yet conclusive. The inhibitory receptor TIGIT (also called Vsig9, Vstm3, and WUCAM) competes with the activation receptor DNAM-1 (CD226) for their ligands poliovirus receptor (PVR; CD155) and Nectin-2 (CD112) [216]. It is expressed on several T cell subsets and NK cells, particularly within the tumor microenvironment where it has been associated with NK cell exhaustion. Blockade of TIGIT improved NK cell function, enhancing tumor-specific T cell immunity and tumor control in syngeneic melanoma and colon cancer models [217]. ISGF8 has recently been identified as a new inhibitory checkpoint that can be expressed by tumors and inhibits NK cell activation through KIR3DL2 in humans and Ly49G in mice [218] Blockade of this checkpoint resulted in increased tumor control in multiple syngeneic murine models of lung, melanoma, colon, and breast cancer [218]. An anti-LILRB1 antibody enhanced NK cell killing against multiple myeloma and breast cancer cell lines in vitro [31]. Taken together, multiple inhibitory checkpoints have been identified that can potentially improve anti-tumor NK cell responses.

ALT-803 (also called N-803): IL-15 superagonist was tested in a phase Ib open-label study in combination with nivolumab in patients with advanced NSCLC who had progressive disease on prior systemic therapies ( [219]. They did not find any dose-limiting toxicities, and observed 29% partial responses with a median progression-free survival (PFS) of 9.4 months and median overall survival (OS) 17.4 months, which was promising in a heavily pre-treated population in both patients who were PD-L1 negative and in patients who had disease refractory to prior checkpoint inhibitor therapy. This provided rationale for testing of pembrolizumab with or without ALT-803 versus standard of care chemotherapy in a phase III randomized controlled study for patients who had resistance on first-line immunotherapy (SWOG study Lung-MAP S1800D, NCT05096663). PFS and OS were not significantly different, however there were signals that indicated a sub-population of patients where this approach might preferentially benefit [220]. Correlative studies from the 2018 open-label phase 2b trial showed that treatment with ALT-803 doubled the frequency of NK cells detectable in the periphery, this expansion was more robust than observed in CD8-positive T cells. Furthermore, Ki67 in NK cells was higher as was expression of serum IFN-γ [219]. N-803 was approved as a therapy in combination with BCG for BCG-unresponsive non-muscle invasive bladder cancer, based on the phase II/III QUILT-3.032 study [221]. Altogether, these results support further study of biomarkers predictive of response to N-803 and investigating N-803 as part of novel combinations in lung cancer.

In SCLC, translational efforts demonstrated four distinct transcriptomic subtypes characterized by high expression of ASCL1/NeuroD1 transcription factors, POU2F3 transcription factor, or the “inflamed” subset enriched in immune infiltrates and interferon-gamma-related gene signatures [222]. Notably, the immune high subset is associated with increased responses to anti-PD1 checkpoint inhibitor therapy, and also shows enrichment of NK cells, in addition to T cells and macrophages. *MICA* gene expression was also upregulated in this inflamed subtype, and as discussed earlier, MICA protein can either stimulate or dampen NK cell responses depending on whether it is membrane-bound or soluble [140]. This provides rationale for targeting NK cell inhibitory receptors in SCLC as well as in NSCLC.

In extensive stage small cell lung cancer, the standard of care involves platinum-doublet chemotherapy along with PD-1/PD-L1 checkpoint inhibition. The CASPIAN trial demonstrated that the addition Durvalumab to platinum doublet chemotherapy extends median overall survival by about 2.7 months (13.0 vs. 10.3 months), hence there is opportunity to mobilize a deeper immune response to improve outcomes [223]. Small cell lung cancer lines and fresh tumor samples have demonstrated decreased expression of MHC-1 which may suggest an innate vulnerability to NK cell mediated killing [224]. Furthermore, xenograft mouse models deficient in NK cell activity develop metastasis more frequently than those with intact NK cell activity suggesting that NK cells play role in preventing metastatic dissemination [225]. However, as discussed above, other NK cell suppressive signals can constrain their activity. The phase 2 MOZART trial is evaluating the addition of Durvalumab and Monalizumab to platinum-doublet chemotherapy in first-line extensive stage SCLC followed by maintenance treatment, and currently enrolling (NCT05903092) [226].

#### NK cell therapy

Chimeric antigen receptors (CAR) have initially been used in T cells to target tumor antigens in B cell lymphomas and multiple myeloma for which it has received FDA approval in 2017, but this type of therapy has not yet been successful in solid tumors [227]. Adoptive transfer of allogenic NK cells has been successful in hematological malignancies without causing severe complications [228]. Engineered NK cells show promise and a potential “off-the-shelf” cellular therapy product, with less toxicity than T cell-based therapies, however NK cell activity and persistence remain key limitations [229]. Strategies to overcome these challenges, including precise engineering approaches designed to limit toxicities and enhance NK persistence and cytotoxicity, were comprehensively discussed in a recent review by Laskowski, et al. [229].

CARs have been introduced in allogenic NK cells and have shown success in hematological malignancies without severe side effects, such as cytokine release syndrome, which has been observed in CAR T cell therapies [229, 230]. Grote, et al., generated Adapter Chimeric Antigen Receptor (AdCAR)-Engineered NK-92 Cells as an “off the shelf” product to target lymphoma cell lines and patient-derived lymphoma cells [231]. They employed biotinylated antibodies as adapter molecules to redirect CAR-NK-92 cells, which could be modified to target multiple tumor types, and later utilized this system to target breast, kidney, colorectal, and melanoma cell lines that were derived from bone metastases [232].

The Rezvani group designed a CD19 CAR-NK (CAR19/IL-15) product from cord blood NK cells that showed excellent activity in B cell lymphoma, and identified cord blood unit features (decreased nucleated red blood cell count and short collection to cryopreservation time) that improved yield of functional NK cells [233]. Of note, they did not observe instances of cytokine release syndrome, neurologic toxicities, or graft versus host disease [233]. Investigators used a similar approach to design a CD70/IL-15 CAR-NK product from optimally collected/preserved cord blood units, which was tested in a basket trial for patients with treatment-refractory hematologic malignancies (NCT05092451). This CAR-NK product could be adapted for solid tumors, as CD70 is also highly expressed on other malignancies including renal cell carcinoma, esophageal carcinoma, mesothelioma, and lung carcinomas [234].

CAR-NK therapy is currently being explored for several tumor types, including ovarian carcinoma, prostate cancer and lung cancer [229]. Moreover, several strategies to engineer NK cells for improved allogeneic therapies are being investigated [229]. Lentiviruses are currently the most widely used method to engineer lymphocytes, but it has the risk for secondary malignancies as has been recently described for CAR T cell therapy [235]. However, more precise editing strategies such as CRISPR/Cas9 may overcome these risks. While in various stages of development, engineered NK cells may prove valuable cellular immunotherapies for (specific) malignancies, and has been reviewed previously [169].

NK cell infusion along with checkpoint inhibitor therapy has been investigated in NSCLC. A pilot study including 20 patients with metastatic NSCLC with disease progression after first-line platinum-based chemotherapy were treated with sintilimab (checkpoint inhibitor, anti-PD-1 antibody) and autologous NK cells [236]. PBMCs were collected 14 days prior to NK and sintilimab infusion, and NK cells were expanded using media containing 5% autologous plasma, 600 U/mL IL-2, and 10 ng/mL IL-15, and 1 ug/mL OK432. After expansion, 3 × 10^9 NK cells were infused every 3 weeks in conjunction with sintilimab. They reported median PFS of 11.6 months with 45% overall response rate. Limitations of this study include small sample size, and evolution of NSCLC standard therapy that now incorporate checkpoint inhibitors in the first-line setting [237–239].

A similar phase IB trial evaluated the combination of cytokine-induced killer cells (CIK), Sintilimab, and platinum-based chemotherapy in the first-line setting for stage IIIB-IV NSCLC [240]. CIK cells are comprised of CD3^−^/CD56^+^ natural killer (NK) cells and CD3^+^/CD56^−^ cytotoxic T cells expanded by cytokines. PBMCs were collected from patients on day 0 of the cycle and cultured in media containing anti-CD3 antibodies and (IFN)-γ. IL-2 and (IFN)-γ were added every 4 days. On day 13 CIK cells were harvested and analyzed for phenotype and cytotoxicity. Chemotherapy and Sintilimab were administered on day 1 of each cycle and CIK cells on day 13 (total count ≥ 1 × 10^10^) for 4 cycles followed by maintenance Sintilimab with or without pemetrexed (depending on histology). 34 patients were treated with an ORR of 82.4% and DCR of 100%. Median PFS was 19.3 months and median OS was not reached. Grade 3 or higher treatment related adverse events were seen in 64.7% of patients and grade 3 or higher immune related adverse events were seen in 11.8% of patients. Although this trial is limited by a small sample size, the impressive response rates do warrant further investigation in a larger randomized controlled trial. A phase 2 study is currently ongoing. (NCT04836728).

Work from Multhoff et al. has demonstrated that heat shock protein 70 (hsp70) is preferentially expressed on the surface of NSCLC cells and that chemo-/radiotherapy increases expression density [241]. NK cells can be stimulated to recognize this protein to mount an immune response. In a phase 2 trial patients with unresectable stage III NSCLC who had elevated serum exosomal hsp70 levels were randomized to either 4 cycles of autologous NK cells (> 1.04 × 10^8^) after chemoradiotherapy or chemoradiotherapy alone [241]. Peripheral lymphocytes were isolated and incubated with TKD-peptide (partial hsp70 sequence) and IL2 prior to infusion. Infusions were administered every 2–6 weeks up to 4 times. The number of reinfused NK cells increased between the first and third reinfusion cycles and remained elevated after the fourth. 16 patients were included (8 in the control arm and 8 in the intervention arm). The estimated 1-year PFS was 67% in the intervention arm and 33% in the control arm. The trial had difficulty accruing and was terminated after the PACIFC trial regimen was approved and Durvalumab became the standard of care after chemoradiotherapy [242]. It remains to be seen whether the combination of autologous NK cells and PD1 checkpoint inhibition after chemoradiation could improve outcomes.

Allogeneic NK cellular therapy has also been explored in NSCLC. A phase 1/2 trial was conducted evaluating the combination of Pembrolizumab and allogeneic NK cells as compared with Pembrolizumab monotherapy in patients with recurrent stage IIIB or IV PDL1 ≥ 1% NSCLC [243]. Donors were chosen so that there would be genotyping mismatch between the KIR of allogenic donors and the HLA class I molecules of patients. PBMCs were isolated from donors and a synergist was used to activate and expand NK cells along with IL2. In the intervention group, patients were treated with 1 to 3 courses of allogeneic NK cells with 1 treatment course including 2 cycles in a 28-day period. Pembrolizumab was administered every 3 weeks in both groups. 109 patients were included with 55 in the intervention group and 54 in the control group. The ORR for the Pembrolizumab with NK cell therapy group was 36.4% vs. 18.5% in the Pembrolizumab monotherapy group. Both PFS and OS were longer in the intervention group. There was no significant difference in adverse events between the two groups and there were no grade 4 adverse events. While the results of this trial do suggest clinical activity, this study is limited in that PD1 checkpoint inhibition is currently incorporated into front-line treatment [237–239]. A phase 1/2a trial (NCT04616209) is currently being conducted evaluating the efficacy of PB103 allogeneic NK cells derived from healthy donors in recurrent stage IIIB/IV NSCLC, and estimated to complete enrollment by the end of 2024.

Newer combinations of adoptive NK cell therapy and novel immune-activating antibodies represent alternative approaches that could be more effective than standard checkpoint inhibitors. Nieto, et al., reported excellent ORR (including 67% complete responses) in patients with treatment-refractory Hodgkin lymphoma (HL) treated with the combination of a CD30-CD16A bispecific antibody (AFM13) and cord-blood-derived NK cells [244]. The NK cells were pre-complexed with AFM13 prior to infusion. Patients received lymphodepletion regimens, followed by AFM13-NK cell infusion, and then received 3 additional weekly infusions of the AFM13 bispecific antibody [244]. Of note, patients on this trial had disease progression after standard HL therapies including anti-PD1 checkpoint inhibitors. Bispecific engagers are already approved in SCLC (the DLL3-CD3 BiTE tarlatamab) [128], and thus this could be an intriguing approach for future investigation in lung cancer in which standard of care includes checkpoint inhibitors.

## Conclusions

NK cells are crucial in tumor immune responses, both as immunomodulators and effectors of direct cytolytic activity. Unleashing inhibitory checkpoints on NK cells with bispecific antibodies and/or engineered NK cells are promising approaches in improving efficacy and durability. These approaches may combat both primary and acquired resistance to current immunotherapy. Further investigation of tumor-specific and microenvironment-specific effects on NK cells will provide the necessary insight to guide development of new immunotherapy targets and rationally designed combination therapies, including combinations of bispecific antibodies, checkpoint inhibitors, and NK cellular therapies.

**Table 1:** NK cell-based therapies in lung cancer

Mechanism	Intervention	Phase	Results	NCT
IL15-superagonist	ALT-803 + Nivolumab in Advanced NSCLC after progression on prior therapy	1b	ORR: 29% PFS: 9.4 months OS: 17.4 months	NCT02523469
	ALT-803 + Pembrolizumab in Advanced NSCLC after progression on prior therapy vs. Standard of Care	2/3	No significant difference in PFS/OS	NCT05096663
NKG2A Inhibition	Monalizumab with Durvalumab in Advanced Solid Tumors after progression on prior therapy	1/2	NSCLC cohort: ORR 10%, Median PFS: 1.9 months, Median OS: 8.8 months	NCT02671435
	COAST: Consolidative Durvalumab alone or in combination with Monalizumab or Oleculumab after chemoradiation for Stage III NSCLC	2	12 Month PFS D + M: 72.7% D + O: 62.6% D: 33.9%	NCT03822351
	PACIFIC-9: Consolidative Durvalumab alone or in combination with Monalizumab or Oleculumab after chemoradiation for Stage III NSCLC	3	Ongoing	NCT05221840
	NEOCOAST: Neoadjuvant Durvalumab alone or in combination with Monalizumab, Oleculumab, or Danvatirsen in Stage I-IIIA NSCLC	2	pCR: D + M: 10.0% D + O: 9.5% D + Da: 12.5% D: 3.7%	NCT03794544
	NEOCOAST-2: Perioperative Durvalumab + Chemotherapy in combination with Monalizumab, Oleculumab, Volrustomig, or Dato-DXd in Stage II-IIIB NSCLC	2	pCR: D + M: 26.7% D + O: 20.0% D + D-DXd: 34.1%	NCT05061550
	MOZART: Monalizumab + Durvalumab + Chemotherapy for Extensive-Stage Small Cell Lung Cancer, 1st line	2	Ongoing	NCT05903092
Autologous NK Cells	Autologous NK cells + Sintilimab in Advanced NSCLC after progression on prior therapy	2	ORR: 45% PFS: 11.6 months OS: 17.7 months	NCT03958097
	Autologous CIK Cells + Sintilimab + Chemo in Advanced NSCLC, 1st line	1b 2	ORR: 82.4% PFS: 19.3 months Ongoing	NCT03987867 NCT04836728
	Autologous NK cells after chemoradiation for Stage III NSCLC	2	1Y PFS: Experimental: 67% Control: 33%	NCT02118415
Allogeneic NK Cells	Allogeneic NK cells + Pembrolizumab in advanced NSCLC after progression on prior therapy	1/2	ORR: *P* + NK: 36.4% P: 18.5%	NCT02843204
	Allogeneic PB103 NK cells in advanced disease	1/2a	Ongoing	NCT04616209
BiKE	AFM24	1/2a	NSCLC cohort (10 patients): 1 PR, 4 SD	NCT04259450
	AFM24 + Atezolizumab in Advanced EGFR wt solid tumors after progression on prior chemo and ICI	1/2a	NSCLC cohort (15 patients): 1 CR, 3 PR, 7 SD	NCT05109442
TriKE	DF9001 in Advanced EGFR expressing solid tumors after progression on prior therapy	1	Ongoing	NCT05597839
CAR-NK Cells	DLL3-CAR-NK Cells in the Treatment of R/R Extensive Stage Small Cell Lung Cancer	1	Not reported	NCT05507593

ORR: overall response rate, PFS: progression free survival, OS: overall survival, D: durvalumab, M: monalizumab, O: oleculumab, da: danvatirsen, Dato-DXd: datopotamab-deruxtecan, P: pembrolizumab, CR: complete response, PR: partial response, SD: stable disease

**Table 2:** NK cell-based therapies in other solid tumors

Mechanism	Intervention	Phase	NCT
KIR2D Inhibition	Lirilumab with Nivolumab or Lirilumab with Nivolumab and Ipilimumab in Advanced Refractory Solid Tumors	1/2	NCT01714739
	Perioperative Lirilumab with Nivolumab in Squamous Cell Carcinoma of the Head & Neck	2	NCT03341936
IL15-superagonist	ALT-803 in patients with Advanced Solid Tumors	1	NCT01727076
	Intravesicle BCG + ALT-803 in BCG Unresponsive Non-Muscle-Invasive Bladder Cancer	3	NCT03022825
NKG2A Inhibition	Durvalumab + Monalizumab in Non-Muscle Invasive Bladder Cancer	2	NCT06503614
	Monalizumab + Volrustomig in patients with MSI and/or ddMMR Metastatic Cancer	2	NCT06152523
	Monalizumab + Trastuzumab in Metastatic HER2 + Breast Cancer	2	NCT04307329
	Monalizumab + Cetuximab vs. Placebo + Cetuximab in Recurrent of Metastatic Head & Neck Cancer	3	NCT04590963
Autologous NK Cells	SNK01 (Autologous NK cells) alone or in combination with Pembrolizumab or Avelumab in Refractory Unresectable or Metastatic Cancer	1	NCT03941262
	Memory Cytokine Enriched NK (M-ceNK) cells in Locally Advanced or Metastatic Solid Tumors	1	NCT04898543
	Autologous DC and NK cells + Anti-PD1 antibody in the Treatment of Digestive Carcinomas	2	NCT05461235
Allogeneic NK Cells	SNK02 (Allogeneic NK cells) in Refractory Cancer	1	NCT05990920
	Allogeneic NK cell therapy in Advanced Hepatocellular Carcinoma	1/2	NCT04162158
	SMT-NK (Allogeneic NK cells) + Pembrolizumab vs. Pembrolizumab alone in Advanced Biliary Tract Cancer	2/3	NCT05429697
BiKE + Autologous NK Cell	AFM24 + SNK01 in patients with metastatic EGFR Expressing Tumors	1/2a	NCT05099549
BiKE	Intratumoral Microdose Administration of PBA-0405 in Solid Tumors	0	NCT06273852
TriKE	DF1001 in Advanced HER-2 expressing Solid Tumors	1/2	NCT04143711
ANKET/Tetra-specific NK Cell Engager	IPH6501in R/R B-Cell Non-Hodgkin Lymphoma	1/2	NCT06088654
CAR NK and Engineered NK Cells	**NKG2D-Ligand CAR-NK Cells in patients with** Metastatic Solid Tumors	1	NCT03415100
	Intraperitoneal CD16-Ligand CAR-NK cells + IL2 + B7-H3 inhibitor in advanced R/R gynecologic malignancies	1	NCT04630769
	**PD-L1 CAR-NK Cells + Pembrolizumab + N-803 for Recurrent/​Metastatic Gastric or Head and Neck Cancer**	2	NCT04847466
	PDL-1 t-haNK + N-803 + Bevacizumab for Recurrent or Progressive Glioblastoma	2	
	Mesothelin-Ligand CAR-NK Cells in Epithelial Ovarian Cancer	1	NCT03692637
	5T4-Ligand CAR-NK Cells in patients with Advanced Solid Tumors	1	NCT05194709
	CLDN6/GPC3/Mesothelin/AXL-CAR-NK in Patients with CLDN6/GPC3/Mesothelin/AXL-positive Advanced Solid Tumors	1	NCT05410717
	TROP-2 CAR NK Cells in patients with Advanced Solid Tumors	1	NCT06066424
	NY-ESO TCR-NK in Advanced Synovial Sarcoma and Myxoid/Round Cell Liposarcoma	1	NCT06083883
	PRAME TCR-NK in Recurrent/Refractory Melanoma (PRAMETIME-Mel)	1	NCT06660420

## Data Availability

No datasets were generated or analysed during the current study.

## Abbreviations

ADAM10: A disintegrin and metalloproteinase 10
ADCAR: Adapter chimeric antigen receptor
ADCC: Antibody-dependent cellular cytotoxicity
ALK: Anaplastic lymphoma kinase
ALT: Alanine transaminase
ANKET: Antibody-based NK cell Engager Therapeutics
AP-NK: Antigen presenting NK cells
BoM: Bone metastases
BrM: Brain metastases
CAR: Chimeric antigen receptor
CCR2: C-C chemokine receptor 2
CCR7: C-C chemokine receptor type 7
CEACAM-1: Carcinoembryonic antigen-related cell adhesion molecule 1
CIK: Cytokine-induced killer cells
cNK: Cytolytic NK cells
CNS: Central nervous system
CTLA4: Cytotoxic T-lymphocyte-associated protein 4
CXCR4: C-X-C chemokine receptor type 4
CXCR6: C-X-C Motif chemokine receptor 6
CX3CL1: Chemokine (C-X3-C motif) ligand 1
DCs: Dendritic Cells
DNAM-1: DNAX accessory molecule-1
EGFR: Epidermal growth factor receptor
FGL1: Fibrinogen-like protein 1
FLT3: FMS-like tyrosine kinase 3
GZMB: Granzyme B
HIF1α: Hypoxia-inducible factor 1-alpha
HMGB1: High mobility group box 1
HSP70: Heat shock protein 70
IDO1: Indoleamine 2,3-dioxygenase 1
ILC1: Type 1 Innate Lymphoid cells
KIR3DL2: Killer cell immunoglobulin like receptor
KLRC1: Killer Cell Lectin-Like Receptor Subfamily C, Member 1
LAG3: Lymphocyte activation gene 3
LLC: Lewis lung carcinoma
MICA/MICB: MHC Class I chain-related protein A and B
MHC: Major histocompatibility complex
NKh: Helper NK cells
NK: Natural Killer Cells
NKG2A: Natural killer group 2 member A
NKG2D: Natural killer group 2, member D
NKreg: Regulatory NK cells
NSCLC: Non-small cell lung cancer
ORR: Objective response rate
OS: Overall survival
PB-NK: Peripheral blood NK cells
PBMCs: Peripheral blood mononuclear cells
PD-1: Programmed cell death protein 1
PFS: Progression-free survival
PVR: Poliovirus receptor
RANK: Receptor activator of nuclear factor-kappa B
sMIC: Soluble MIC
scRNAseq: Single cell RNA sequencing
SCLC: Small cell lung cancer
SLAM: Signaling lymphocytic activation molecule
SREs: Skeletal-related events
STING: Stimulator of interferon genes
TCGA: The cancer genome atlas
TGBF: Transforming growth factor beta
TIGIT: T cell immunoreceptor with immunoglobulin and ITIM domain
TIM-3: T-cell immunoglobulin and mucin domain 3
TME: Tumor microenvironment
TRAMP model: Transgenic adenocarcinoma of the mouse prostate model
TriKEs: Tri-specific killer engagers
TrNK: Tissue-resident NK cells
ULPB1/ULPB2/ULPB3: UL16 binding protein 1 and 2 and 3
